# Artificial intelligence in neurosurgical decision-making: promise and peril in the United States practice

**DOI:** 10.1097/MS9.0000000000004636

**Published:** 2026-01-14

**Authors:** Tirath Patel, Hamza Yousuf Ibrahim, Sana Sohrab, Muhammad Taha Altaf, Bhumi Daishik Patel, Nikhilesh Anand

**Affiliations:** a Department of Neurosurgery, Trinity Medical Sciences University School of Medicine, Ratho Mill, Kingstown, Saint Vincent and the Grenadines; b Department of Neurosurgery, Jinnah Medical and Dental College, Karachi, Pakistan; c Department of Neurosurgery, Dow University of Health Sciences, Karachi, Pakistan; d Department of Neurosurgery, Ameer-ud-Din Medical College, Post Graduate Medical Institute (PGMI), Lahore, Pakistan; e Department of Neurosurgery, Windsor University School of Medicine, Cayon, Saint Kitts and Nevis; f Department of Medical Education, University of Texas Rio Grande Valley, Edinburg, Texas, USA

**Keywords:** artificial intelligence, clinical decision-making, FDA approval, neurosurgery, rural health

## Abstract

The rapid integration of artificial intelligence (AI) into neurosurgical practices in the United States is transforming how diagnoses are interpreted, how surgical plans are developed, and how guidance is provided during operations as clinical needs continue to grow. Models based on radiomics and the Food and Drug Administration approved tools for tumor segmentation, aneurysm identification, and spinal navigation are showing enhanced accuracy and decreased variability among observers, highlighting AI’s potential for significant change. Nevertheless, there are major concerns about the opacity of black-box models, inaccurate outputs, and the increasing legal uncertainties arising from AI-related errors. Ethical challenges, such as the risk of clinician de-skilling, diminished professional autonomy, and exacerbated inequities in rural areas with limited access to imaging, complicate responsible deployment. This letter emphasizes the necessity for transparent validation processes, oversight led by clinicians, diverse and inclusive datasets, and improved regulatory protections. Requiring AI competency training and nationally reporting adverse events associated with AI are crucial to ensure that AI enhances clinical judgment rather than replacing it, thereby maintaining patient safety, equity, and professional integrity in neurosurgical decision-making.

*To the Editor,*

The rapid adoption of artificial intelligence (AI) in neurosurgical practice has become increasingly relevant in the post-pandemic era, where clinical demands continue to strain the United States (U.S.) surgical systems. Rising case volumes, staffing shortages, and growing diagnostic complexity have driven the integration of AI-driven applications for neuroimaging interpretation, operative planning, and surgical navigation. Radiomics-based models and machine-learning algorithms now enable automated glioma grading, spine surgery planning, and real-time intraoperative guidance. The Food and Drug Administration (FDA) 2023–2024 AI-enabled device approvals highlight how rapidly clinical tools are entering U.S. neurosurgical practice. FDA-cleared segmentation and aneurysm-detection platforms have demonstrated improvements in tumor-margin detection and reductions in observer variability, while AI-assisted spine navigation systems support greater diagnostic precision and hold potential to improve workflow efficiency^[1]^.

However, important challenges raise critical concerns regarding the safe and responsible use of AI. Most neurosurgical AI tools still operate as black-box models, and a tension persists between clinical expectations for interpretability and the limited transparency of current systems. Risks of hallucinations or inaccurate outputs remain concerning for high-stakes surgical decision-making. Integration with electronic health record systems is inconsistent, at times disrupting rather than supporting clinical workflow. Moreover, errors arising from AI-influenced recommendations introduce new medico-legal uncertainty about the shared responsibility among clinicians, hospitals, and developers, underscoring the need for stronger regulatory oversight^[2,3]^.

Growing dependence on these tools risks diminishing clinical autonomy and eroding professional and ethical standards^[4]^, ultimately leading to the de-skilling of physicians and raising serious credibility concerns^[5]^. The FDA requires AI-driven tools for tumor segmentation, hemorrhage detection, or operative planning to be explainable so that surgeons can interpret the rationale behind system recommendations^[6]^. Compounding these issues is a shortage of MRI and CT imaging in rural areas. Low-power AI models, data harmonization, and federated learning approaches are urgently needed. Combined with excessive reliance on AI systems, current limitations make it increasingly difficult for rural practitioners to deliver equitable and context-sensitive care, as reflected in rising medicolegal disputes in these regions. Furthermore, many rural hospitals depend on older CT and MRI systems that lack the resolution and software compatibility required for modern AI-based analysis^[7]^.

AI in neurosurgery, therefore, requires a careful balance between innovation and accountability. Transparent validation protocols, clinician-led oversight, and strict ethical safeguards are essential to ensure safe implementation. Mandatory AI-competency training within neurosurgical education, supported by advanced workshops, should be prioritized. A national registry for AI-associated adverse events, along with broader inclusion of diverse patient datasets, especially from underserved areas, will help mitigate algorithmic bias and improve generalizability. Future research must prioritize prospective validation of AI models in diverse U.S. populations. Ultimately, AI should augment, not replace, clinical judgment, ensuring that technological advancement remains aligned with core principles of patient safety, equity, and professional integrity. A balanced, ethically grounded deployment strategy is essential for AI to truly strengthen the U.S. neurosurgical care.

This manuscript is made compliant with the TITAN checklist to ensure transparency in the reporting of AI^[8]^.

## Data Availability

No new dataset was generated during the study.

## Research registration unique identifying number (UIN)

Not required because the study is a letter to the editor.
